# Supplementary material for: Discovery of fast and stable proton storage in bulk hexagonal molybdenum oxide
Source: Nat Commun. 2023 Dec 15;14:8360. doi: 10.1038/s41467-023-43603-6 (PMC10724264; doi:10.1038/s41467-023-43603-6)
Supplement: Supplementary file 1 — Supplementary Information [file 41467_2023_43603_MOESM1_ESM.pdf]

Supplementary Information

Discovery of fast and stable proton storage in bulk hexagonal molybdenum oxide

Tiezhu Xu[1], Zhenming Xu[1], Tengyu Yao[1], Miaoran Zhang[1], Duo Chen[1], Xiaogang Zhang[1], Laifa Shen[1]*

[1]Jiangsu Key Laboratory of Electrochemical Energy Storage Technologies, College of Material Science and Technology, Nanjing University of Aeronautics and Astronautics, Nanjing 211106, People's Republic of China

Email: Laifa Shen, lfshen@nuaa.edu.cn

Contents

Supplementary Figures

Fig. 1 Characterizations of α-MoO_3 4

Fig. 2 Crystal structure analysis of α-MoO_3. 5

Fig. 3 SEM images of α-MoO_3 nanorods. 6

Fig. 4 TG curves of h-MoO_3 microrods. 7

Fig. 5 TG curves of α-MoO_3 nanorods 8

Fig. 6 XPS spectrum of Mo 3d for h-MoO_3 9

Fig. 7 CV curves of h-MoO_3 electrodes in H_2SO_4 with various pH values 10

Fig. 8 Electrochemical performance of α-MoO_3 electrodes 11

Fig. 9 Cycling stability performance for α-MoO_3 electrodes at 20 A g^{-1} 12

Fig. 10 Kinetic analysis of h-MoO_3 electrodes 13

Fig. 11 SEM images of h-MoO_3 nanoparticles after mechanical milling 14

Fig. 12 Capacitance and GCD curves of nanostructured h-MoO_3 electrodes 15

Fig. 13 Cycling stability performance for nanostructured h-MoO_3 electrodes. 16

Fig. 14 CV curves of h-MoO_3 electrodes at different electrolytes 17

Fig. 15 Volumetric capacitance and areal capacitance of h-MoO_3 electrodes 18

Fig. 16 EIS of h-MoO_3 at different temperatures 19

Fig. 17 The bulk impedance spectrum of α-MoO_3 at different temperatures 20

Fig. 18 Structural schematic of possible binding sites for protons in *h*-$MoO_3 \cdot 0.7H_2O$ (c direction and b direction) 21

Fig. 19 The binding sites of protons in *h*-$MoO_3 \cdot 0.7H_2O$ 22

Fig. 20 The migration pathway of protons in *α*-MoO_3 23

Fig. 21 The distribution of H coordination environments in *h*-$H_{0.5}MoO_3 \cdot 0.7H_2O$ and $HMoO_3 \cdot 0.7H_2O$ structure. 24

Fig. 22 Structural evolution of *α*-MoO_3 25

Supplementary Tables

Table 1. Electrochemical performance comparison of microstructured *h*-MoO_3 with some typical nanostructured pseudocapacitive materials reported 26

Table 2. Electrochemical performance of *h*-MoO_3 and reported electrodes 28

Table 3. ICP-OES results of electrolytes for *h*-MoO_3 and *α*-MoO_3 31

Table 4. Electrochemical performance comparison of our work with various supercapacitors reported. 32

Supplementary References 33

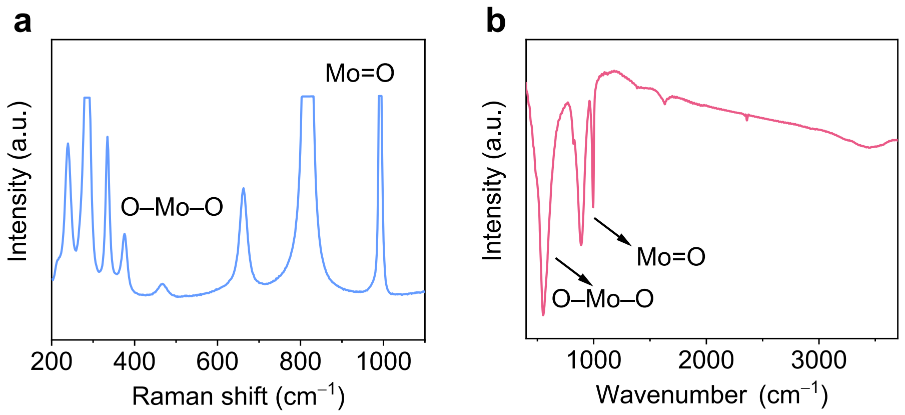

Supplementary Fig. 1 Characterizations of *α*-MoO_3. **a** Raman spectra. **b** FTIR spectra.

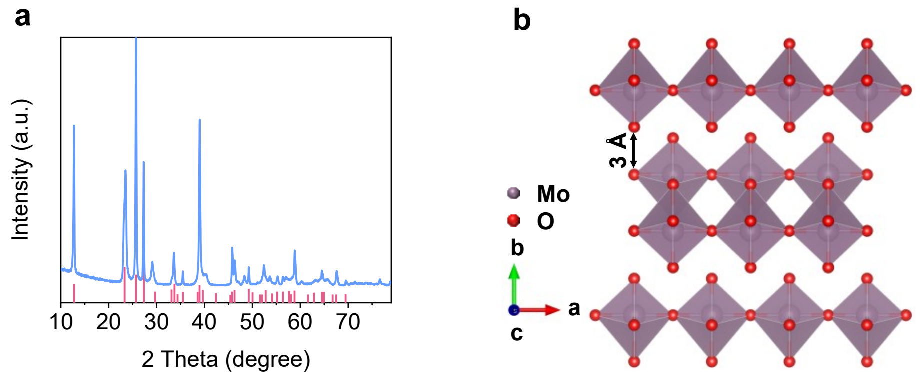

Supplementary Fig. 2 Crystal structure analysis of *α*-MoO_3. a XRD pattern. **b** Crystal structure schematic.

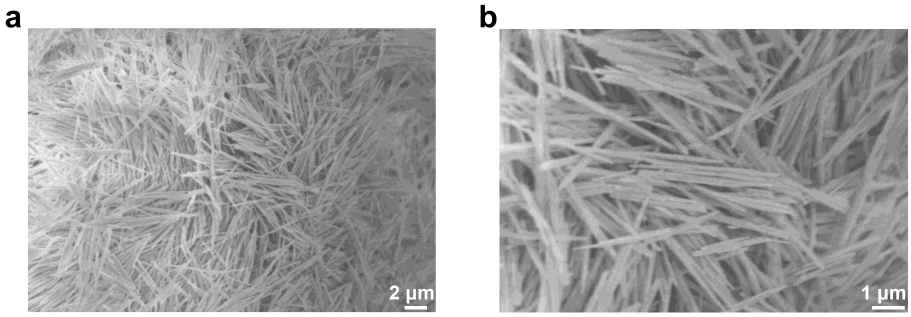

Supplementary Fig. 3 SEM images of α-MoO_3 nanorods. a Scale bars, 2 μm. **b** Scale bars, 1 μm.

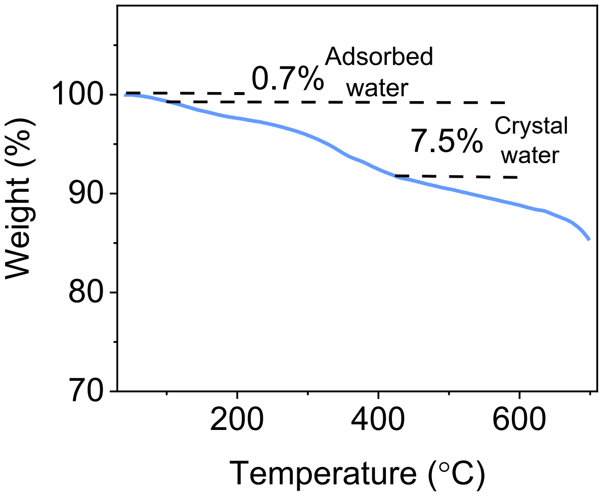

Supplementary Fig. 4 TG curves of *h*-MoO_3 microrods in the temperature range of 25 °C to 700 °C.

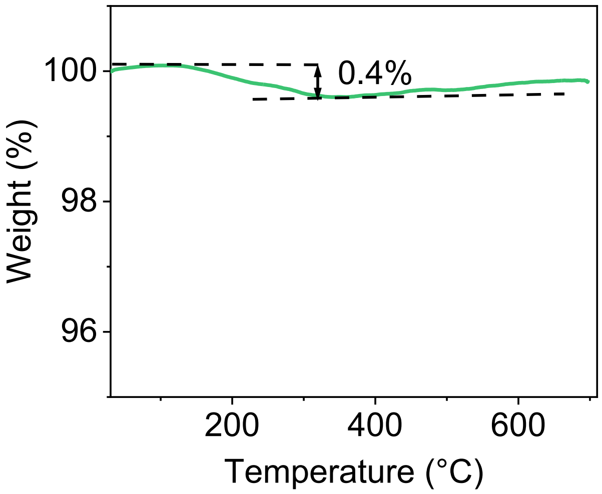

Supplementary Fig. 5 TG curves of α-MoO_3 nanorods.

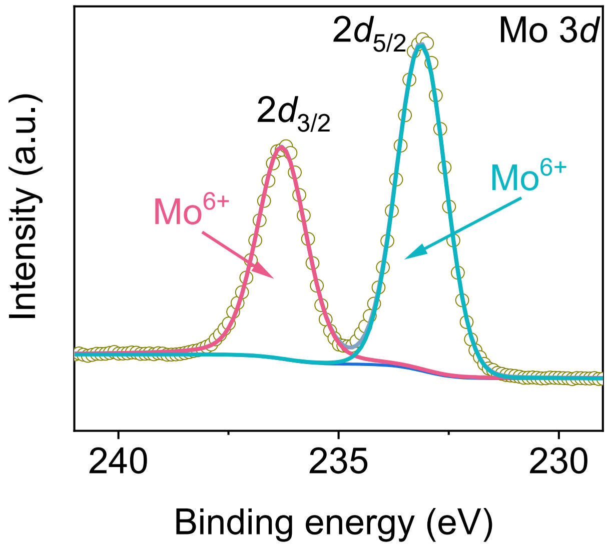

Supplementary Fig. 6 XPS spectrum of Mo 3*d* for *h*-MoO_3.

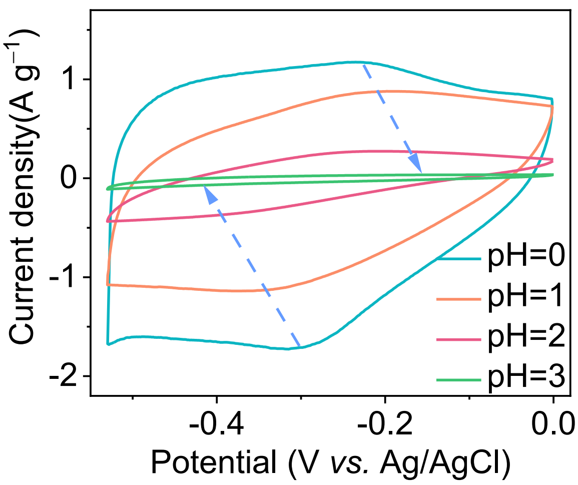

Supplementary Fig. 7 CV curves of *h*-MoO_3 electrodes in H_2SO_4 with various pH values at a scan rate of 2 mV s^{-1}.

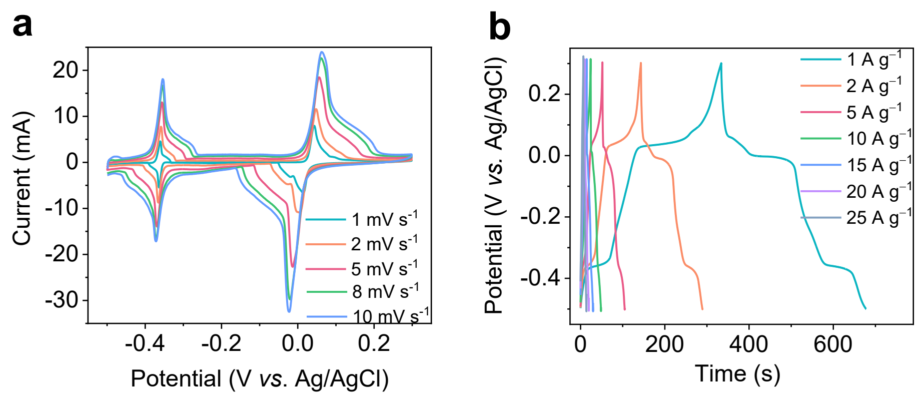

Supplementary Fig. 8 Electrochemical performance of *α*-MoO_3 electrodes. a CV curves of *α*-MoO_3 electrodes at various scan rates. **b** GCD curves of *α*-MoO_3 electrodes at different current densities.

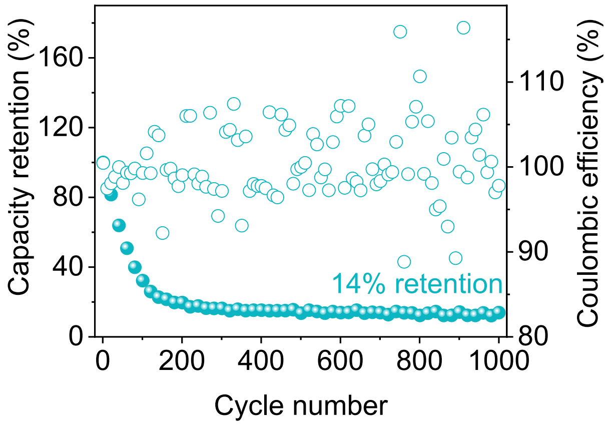

Supplementary Fig. 9 Cycling stability performance for α-MoO_3 electrodes at 20 A g^{-1}.

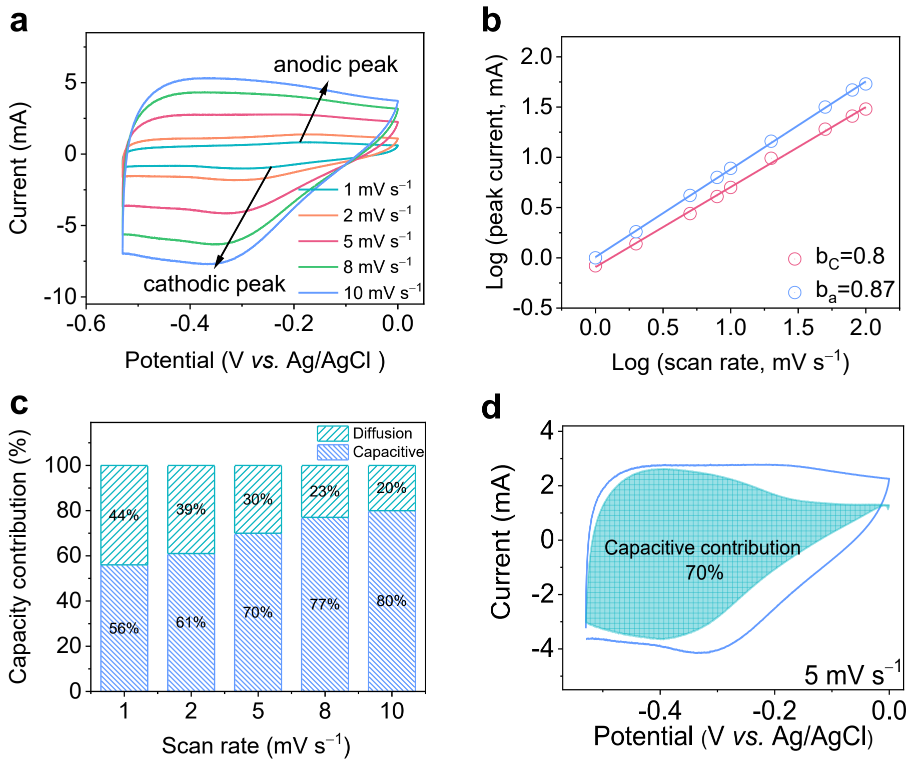

Supplementary Fig. 10 Kinetic analysis of *h*-MoO_3 electrodes. a CV curves and the position of the redox peak at various scan rates. **b** b values corresponded to anodic and cathodic peaks. **c** Capacitive contribution and diffusion contribution at different scan rates between 1 and 10 mV s^{-1}. **d** Capacitive contribution at a scan rate of 5 mV s^{-1}.

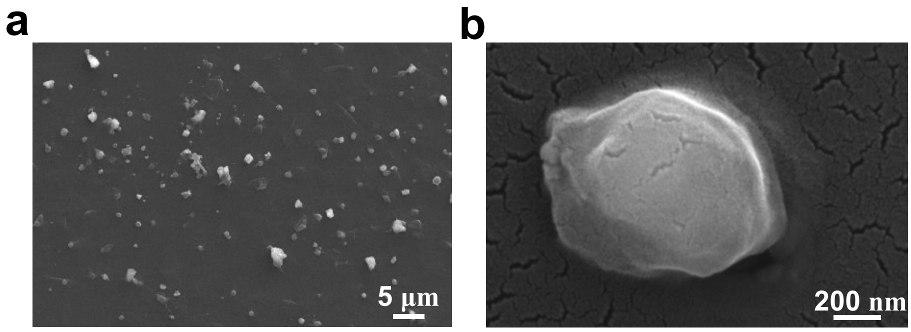

Supplementary Fig. 11 SEM images of *h*-MoO_3 nanoparticles after mechanical milling. **a** Scale bars, 5 μm. **b** Scale bars, 200 nm.

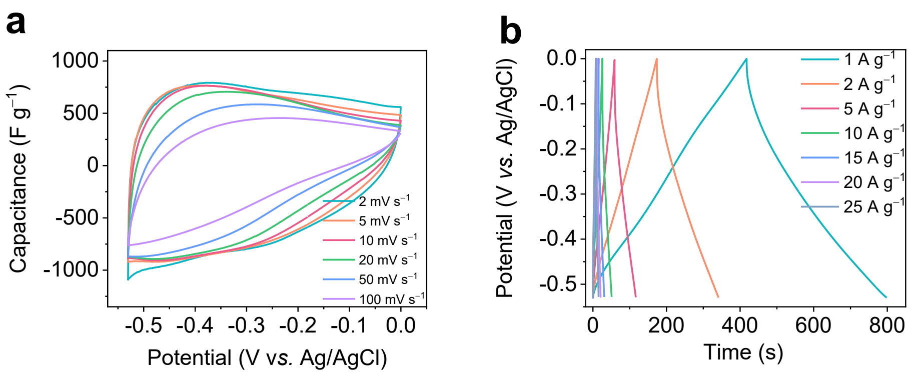

Supplementary Fig. 12 Electrochemical performance of nanostructured *h*-MoO_3 electrodes. a Capacitance of nanostructured *h*-MoO_3 electrodes at various scan rates. **b** GCD curves of nanostructured *h*-MoO_3 electrodes at different current densities.

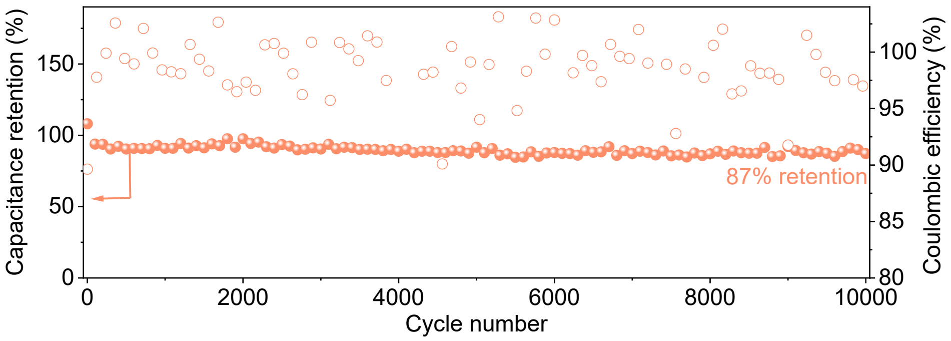

Supplementary Fig. 13 Cycling stability performance for nanostructured *h*-MoO_3 electrodes at 20 A g^{-1}.

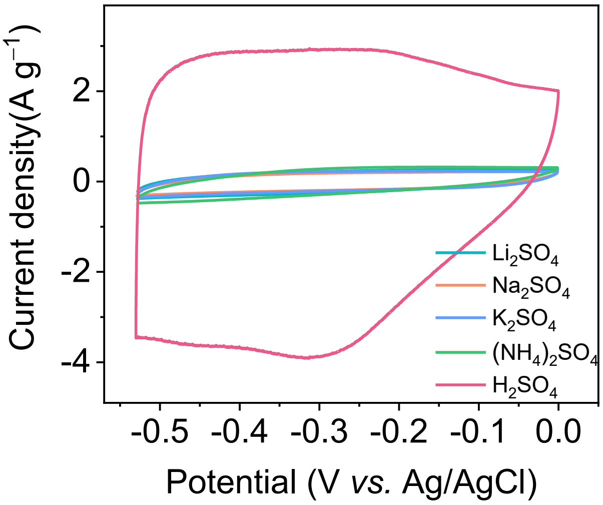

Supplementary Fig. 14 CV curves of *h*-MoO_3 electrodes at different electrolytes at a scan rate of 5 mV s^{-1}.

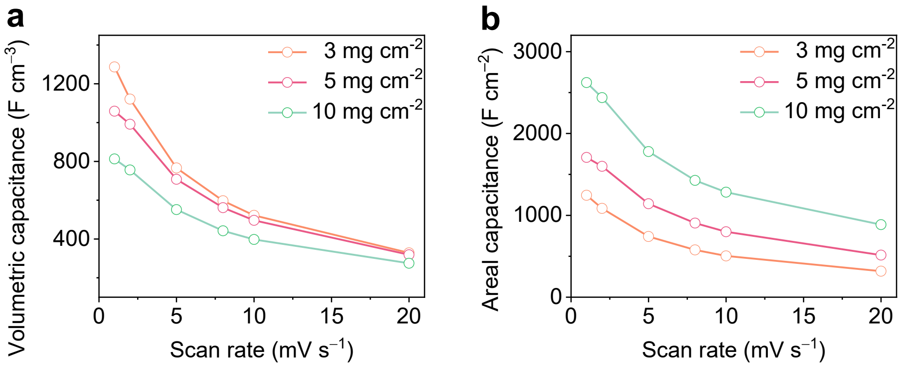

Supplementary Fig. 15 Volumetric capacitance and areal capacitance of *h*-MoO_3 electrodes at different mass loadings. a Volumetric capacitance. **b** Areal capacitance.

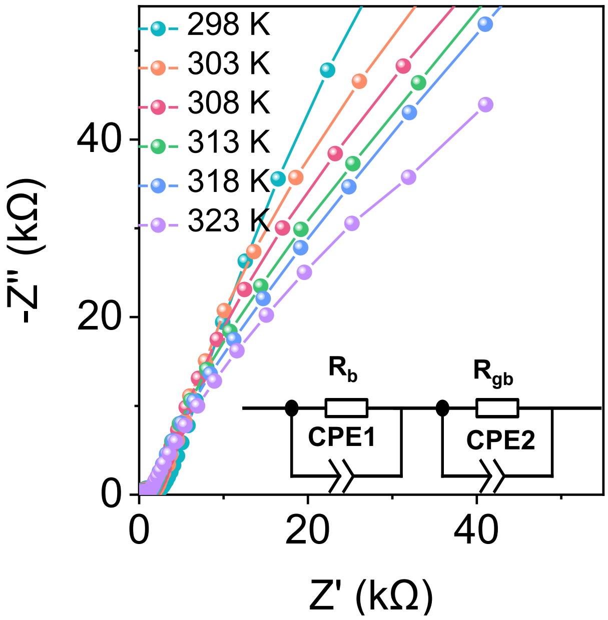

Supplementary Fig. 16 EIS of *h*-MoO_3 at different temperatures (Inset is the equivalent circuit).

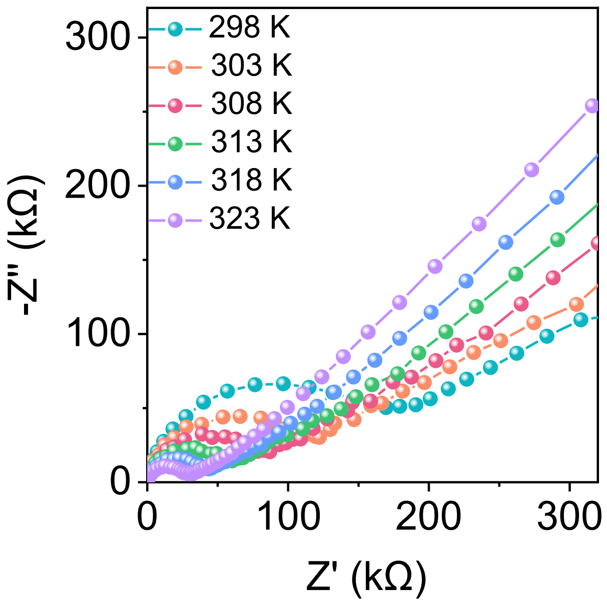

Supplementary Fig. 17 The bulk impedance spectrum of *α*-MoO_3 at different temperatures.

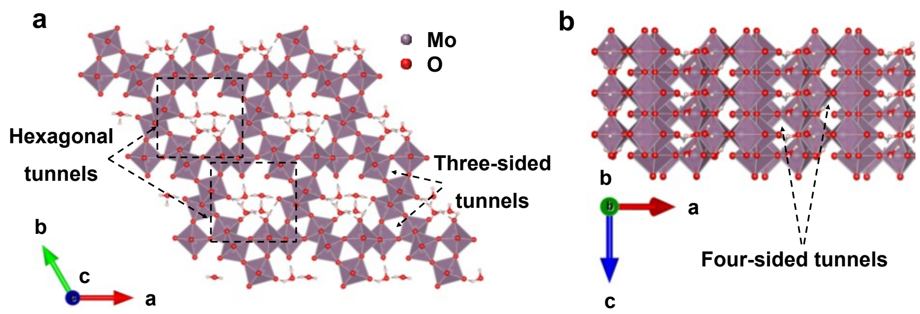

Supplementary Fig. 18 Structural schematic of possible binding sites for protons in *h*-$MoO_3 \cdot 0.7H_2O$. (a) A view down the c direction of the structure. (b) A view down the b direction of the structure.

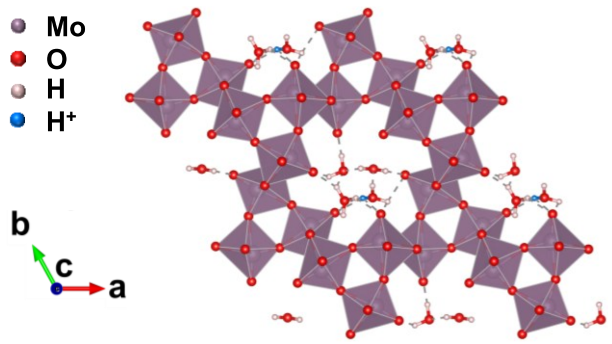

Supplementary Fig. 19 The binding sites of protons in *h*-$MoO_3 \cdot 0.7H_2O$.

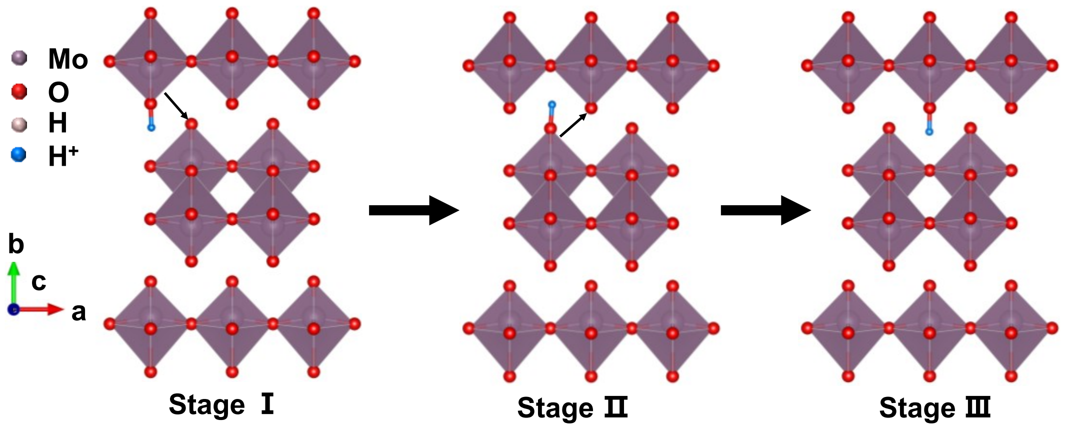

Supplementary Fig. 20 The migration pathway of protons in α-MoO_3.

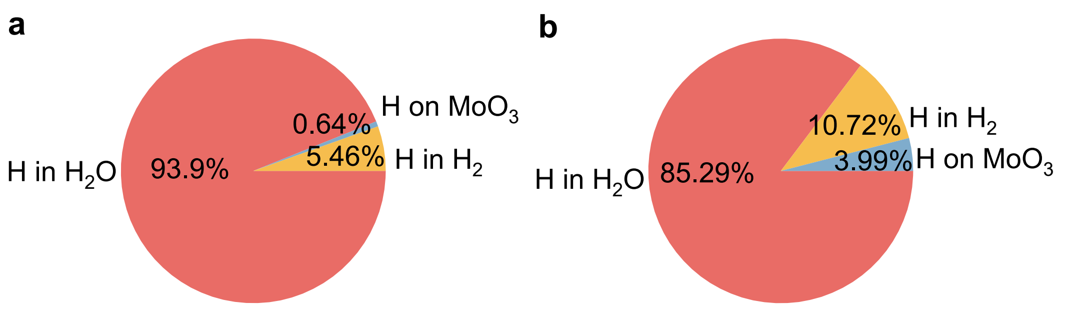

Supplementary Fig. 21 The distribution of H coordination environments from the room temperature AIMD simulations. a *h*-$H_{0.5}MoO_3 \cdot 0.7H_2O$ **b** *h*-$HMoO_3 \cdot 0.7H_2O$.

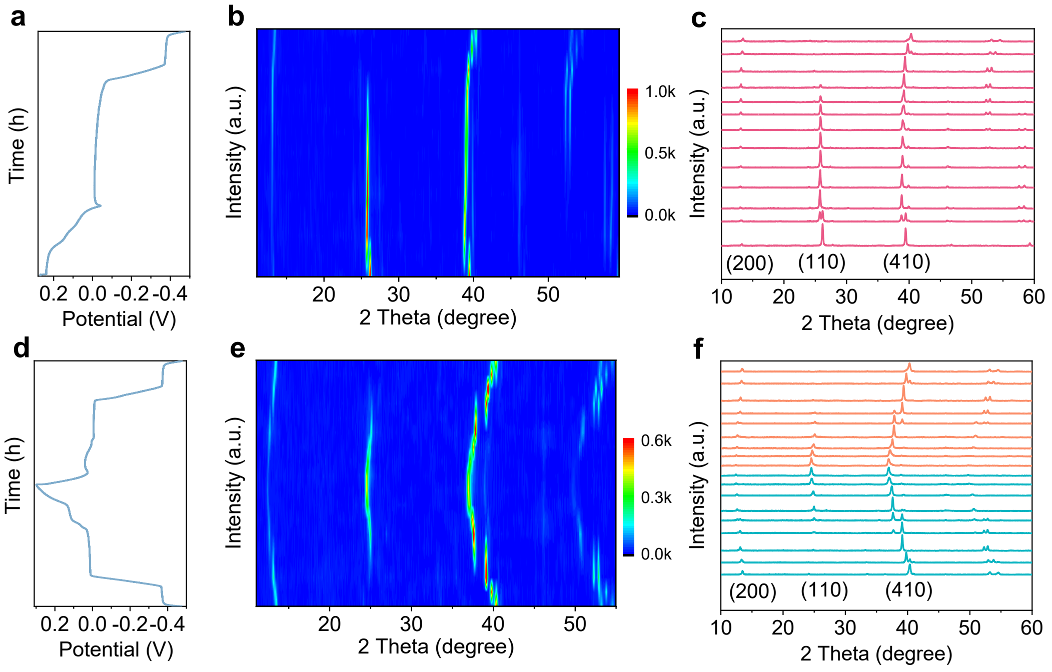

Supplementary Fig. 22 Structural evolution of *α*-MoO_3. a The first discharge curve of *α*-MoO_3 (a current density of 0.03 A g^{-1}). **b** Contour plots of in situ XRD during the first discharge process. **c** In situ XRD patterns of different diffraction peaks during the first discharge. **d** GCD curves of *α*-MoO_3 (the first charge and second discharge processes). **e** In situ XRD patterns during charge and discharge processes. **f** In situ XRD patterns of different diffraction peaks during charge and discharge processes.

Supplementary Table 1 Electrochemical performance comparison of microstructured *h*-MoO_3 with some typical nanostructured pseudocapacitive materials reported in the last five years.

Electrode	Mass loading	Electrolyte	Capacitance ($F\ g^{-1}$)	Rate ($F\ g^{-1}$)	Ref.
G3DTF	—	1 M H_2SO_4	400 (1 mV s^{-1})	113 (100 mV s^{-1})	1
Mo_{132}-DTAB-EEG	—	1 M H_2SO_4	65 (1 mV s^{-1})	~20 (100 mV s^{-1})	2
Cl-MXene	12 mg cm^{-2}	1 M H_2SO_4	304 (2 mV s^{-1})	<100 (100 mV s^{-1})	3
TiNbC MXene	3 mg cm^{-2}	3 M H_2SO_4	381 (2 mV s^{-1})	155 (100 mV s^{-1})	4
Nb@COF	0.1 mg	0.1 M H_2SO_4	367 (2 mV s^{-1})	165 (100 mV s^{-1})	5
Mn_3O_4 NW	9.5 mg	1 M Na_2SO_4	301 (5 mV s^{-1})	<100 (50 mV s^{-1})	6
MoO_3@CNT	2.37 mg cm^{-2}	1 M Na_2SO_4	281 (1 mV s^{-1})	79 (200 mV s^{-1})	7
MnO_2@CNT	1.92 mg cm^{-2}	1 M Na_2SO_4	337 (1 mV s^{-1})	150 (200 mV s^{-1})	7
δ-MnO_2@α-MnO_2	0.9 mg cm^{-2}	1 M Na_2SO_4	348 (5 mV s^{-1})	~200 (100 mV s^{-1})	8
V_2O_5 NBs	0.75 mg cm^{-2}	1 M Na_2SO_4	180 (5 mV s^{-1})	~100 (100 mV s^{-1})	8
$Ti_2V_{0.9}Cr_{0.1}C_2$	—	1 M KOH	553 (2 mV s^{-1})	233 (100 mV s^{-1})	9

T_x					
Li-V_2CT_x	1.3 mg cm^{-2}	1 M LiOH	386 (2 mV s^{-1})	139 (100 mV s^{-1})	10
h-MoO_3	1 mg cm^{-2}	0.5 M H_2SO_4	569 (2 mV s^{-1})	235 (100 mV s^{-1})	This work

Supplementary Table 2 Electrochemical performance of *h*-MoO_3 and reported electrodes

Electrode	Mass Loading (g cm^{-3})	Electrolyte	Potential (V)	Capacitance (F cm^{-3})	Ref.
$Ti_3C_2T_x$ clay	3.6 to 3.8	1 M H_2SO_4	-0.3 to 0.2	900 (2 mV s^{-1})	11
$Ti_3C_2T_x$ hydrogels	3.7 to 4	3 M H_2SO_4	-1.1 to -0.2	1500 (2 mV s^{-1})	12
$Ti_3C_2T_x$ films	—	3 M H_2SO_4	-0.15 to 0.5	676 (—)	13
Cl-$Ti_3C_2T_x$	3.3	1 M H_2SO_4	-0.9 to -0.15	770 (2 mV s^{-1})	3
MXene/rGO	3.1	3 M H_2SO_4	-0.7 to 0.3	1040 (2 mV s^{-1})	14
MXene/CAC	4	3 M H_2SO_4	-0.7 to 0.3	1245 (1 A g^{-1})	15
1T MoS_2	—	1 M H_2SO_4	-0.15 to 0.65	650 (—)	16
10-Gr/m-MoS_2	2	1 M Na_2SO4	-0.2 to 0.8	1431 (1 A g^{-1})	17
2H-1T MoS_2	2.5	6 M KOH	-1 to 0	685 (1 mV s^{-1})	18
1T MoS_2	2.5	1 M Li_2SO_4	-1 to 0.2	1055 (5 mV s^{-1})	19
RuO_2/GO	2.6	1 M H_2SO_4	0 to 1	1485 (0.1 A g^{-1})	20
RuO_2/MXene	—	PVA-KOH	0 to 0.6	864 (1 mV s^{-1})	21
$Ni(OH)_2$	1.1	3 M KOH	0 to 0.5	1562 (10 mA cm^{-2})	22
δ-MnO_2	—	1 M Na_2SO_4	0 to 0.8	922 (5 mV s^{-1})	23
MnO_2/MXene/rGO	—	1 M Na_2SO_4	0 to 0.8	851 (0.25 mA	24

				cm^{-3})	
ANFs/BP-MnO_2	0.88	1 M Na_2SO_4	0 to 0.8	156 (0.25 A cm^{-3})	25
m-WO_{3-x}-C-s	3.3	2 M H_2SO_4	-0.2 to 0.8	340 (1 mV s^{-1})	26
WO_{3-x}	0.74	1 M H_2SO_4	-0.2 to 0.7	125 (2 mV s^{-1})	27
SCNT/$W_{18}O_{49}$ NWs	0.83	1 M $AlCl_3$	-0.4 to 0.6	459 (2 mA cm^{-2})	28
MTB-4	—	1M KOH	0 to 0.8	1172 (0.1 mA cm^{-2})	29
MoN	2.2−3	1 M H_2SO_4	0 to 0.8	928 (2 mV s^{-1})	30
VN	—	1 M KOH	-1 to -0.4	1300 (5 mV s^{-1})	31
VNNDs/CNSs	2.1	1 M KOH	-1 to 0.1	1204 (1.1 A cm^{-3})	32
Ni-HAB MOFs	1.8	1 M KOH	-0.75 to -0.25	760 (0.2 mV s^{-1})	33
COF/rGO	0.84 to 0.68	1 M H_2SO_4	0 to 1	237 (—)	34
TALP	1.8	0.5 M K_2SO_4	-0.2 to 0.4	732 (2 mV s^{-1})	35
rGO/EGM	0.94	$EMIMBF_4$/PVDF-HFP	0 to 4	203 (1 A g^{-1})	36
rGO/SWCNT	1.6	1 M H_2SO_4	0 to 0.9	407 (114 mA cm^{-3})	37
rGO/PPD	1.6	1 M H_2SO_4	0 to 0.8	711 (0.5 A g^{-1})	38
PANI/rGO	1.5	1 M H_2SO_4	0 to 0.8	800 (0.1 A g^{-1})	39
PANI/CCG	1.3	1 M H_2SO_4	0 to 0.9	572 (5 A g^{-1})	40
PPy/rGO	1.6	6 M KOH	-1 to 0	416 (1 A g^{-1})	41

FeSN-C	—	6 M KOH	-0.85 to 0.25	1320 (0.1 A g^{-1})	42
D-SCN	—	2 M KCl	-1 to 0	458 (0.1 A g^{-1})	43
N, P, S-HCS	—	6 M KOH	-1 to 0	219 (0.5 A g^{-1})	44
NSC	1.2	6 M KOH	-0.8 to 0.3	355 (0.5 A g^{-1})	45
CoDC	0.97	6 M KOH	-0.9 to 0.1	262 (1 A g^{-1})	46
h-MoO_3	3.1	0.5 M H_2SO4	-0.53 to 0	1750 (2 mV s^{-1})	This work

Supplementary Table 3 ICP-OES results of electrolytes for *h*-MoO_3 and *α*-MoO_3 after 1000 cycles.

Electrode	*h*-MoO_3	*α*-MoO_3
Mo content (mg L^{-1})	69.9	123

Supplementary Table 4 Electrochemical performance comparison of our work with various supercapacitors reported in the last five years.

Electrode	Electrode loading (mg)	Electrolyte	Max Energy density (Wh kg^{-1})	Max power density (kW kg^{-1})	Ref.
2-GCE//AC	3:1	3 M H_2SO_4	23.4 (3.9 kW kg^{-1})	21.1 (10 Wh kg^{-1})	47
RuO_2//Hex-Aza-COF-3	9.6:8	1 M H_2SO_4	23.3 (0.67 kW kg^{-1})	—	48
1@$Ti_3C_2T_x$//ACL	1:0.72	2 M H_3PO_4	32.2 (2.4 kW kg^{-1})	12 (19.7 Wh kg^{-1})	49
$W_{18}O_{49}$/$Ti_3C_2T_x$//RuO_2@CC	2.4:1.2	1 M H_2SO_4	29.6 (0.75 kW kg^{-1})	7 (24.1Wh kg^{-1})	50
MWCNT/RuO_2//WO_3/$Ti_3C_2T_x$	—	1 M H_2SO_4	27.2 (0.75 kW kg^{-1})	—	51
AC//2-CPE	—	0.5 M H_2SO_4	16.1 (1.7 kW kg^{-1})	10 (10.8 Wh kg^{-1})	52
PYT/GN 4–5//A-$Ti_3C_2T_x$	1.7:1.5	1 M H_2SO_4	18.4 (0.7 kW kg^{-1})	7 (13.2 Wh kg^{-1})	53
Ni–S/1d-Ti_3C_2//d-Ti_3C_2	2.1:1.3	6 M KOH	20 (0.5 kW kg^{-1})	10 (5.1Wh kg^{-1})	54
STO//STO	0.45:0.45	3 M KOH	27.8 (0.3 kW kg^{-1})	19.2 (—)	55
MLMO//MLMO	1:1	3 M KOH	34.1 (0.8 kW kg^{-1})	14.5 (19.4 Wh kg^{-1})	56
MnO_2@CNT//	1.3:1	1 M Na_2SO_4	27.8 (0.52 kW kg^{-1})	10 (9.8 Wh kg^{-1})	7

MoO_3@CNT					
A-Ni-MnBMO//FCNT	0.5:1	1 M Na_2SO_4	27.8 (0.49 kW kg^{-1})	11 (9 Wh kg^{-1})	57
$Cu_{0.82}Co_{0.18}$HCF//*h*-MoO_3	1.2:1	0.5 M H_2SO_4	36 (1 kW kg^{-1})	21 (21 kW kg^{-1})	This work

Supplementary References

1. Witomska, S., *et al.* Graphene oxide hybrid with sulfur-nitrogen polymer for high-performance pseudocapacitors. *J. Am. Chem. Soc.* **141**, 482−487 (2019).

2. Pakulski, D., *et al.* Novel keplerate type polyoxometalate-surfactant-graphene hybrids as advanced electrode materials for supercapacitors. *Energy Stor. Mater.* **17**, 186−193 (2019).

3. Chen, H., Wang, H. & Li, C. Mechanically induced nanoscale architecture endows a titanium carbide MXene electrode with integrated high areal and volumetric capacitance. *Adv. Mater.* **34**, 2205723 (2022).

4. Guan, Y., *et al.* Regulating d-band center of Ti_2C MXene via Nb alloying for stable and high-efficient supercapacitive performances. *Small* **34**, 2301276 (2023).

5. Shanavaz, H., *et al.* Niobium doped triazine based covalent organic frameworks for supercapacitor applications. *J. Energy Storage* **67**, 107561 (2023).

6. Sambath Kumar, K., Cherusseri, J. & Thomas, J. Two-dimensional Mn_3O_4 nanowalls grown on carbon fibers as electrodes for flexible supercapacitors. *ACS Omega* **4**, 4472−4480 (2019).

7. Lee, T. H., Pham, D. T., Sahoo, R., Seok, J., Luu, T. H. T. & Lee, Y. H. High energy density and enhanced stability of asymmetric supercapacitors with mesoporous MnO_2@CNT and nanodot

MoO_3@CNT free-standing films. *Energy Stor. Mater.* **12**, 223−231 (2018).

8. Patil, S. J., Chodankar, N. R., Han, Y.-K. & Lee, D. W. Carbon alternative pseudocapacitive V_2O_5 nanobricks and δ-MnO_2 nanoflakes@α-MnO_2 nanowires hetero-phase for high-energy pseudocapacitor. *J. Power Sources* **453**, 227766 (2020).

9. Ma, W., *et al.* A new $Ti_2V_{0.9}Cr_{0.1}C_2T_x$ MXene with ultrahigh gravimetric capacitance. *Nano Energy* **96**, 107129 (2022).

10. Zhang, T., Matthews, K., VahidMohammadi, A., Han, M. & Gogotsi, Y. Pseudocapacitance of vanadium carbide MXenes in basic and acidic aqueous electrolytes. *ACS Energy Lett.* **7**, 3864−3870 (2022).

11. Ghidiu, M., Lukatskaya, M. R., Zhao, M. Q., Gogotsi, Y. & Barsoum, M. W. Conductive two-dimensional titanium carbide 'clay' with high volumetric capacitance. *Nature* **516**, 78−81 (2014).

12. Lukatskaya, M. R., *et al.* Ultra-high-rate pseudocapacitive energy storage in two-dimensional transition metal carbides. *Nat. Energy* **2**, 17105 (2017).

13. Zhang, C. J., *et al.* Transparent, flexible, and conductive 2D titanium carbide (MXene) films with high volumetric capacitance. *Adv. Mater.* **29**, 1702678 (2017).

14. Yan, J., *et al.* Flexible MXene/graphene films for ultrafast supercapacitors with outstanding volumetric capacitance. *Adv. Funct. Mater.* **27**, 1701264 (2017).

15. Zhang, P., Li, J., Yang, D., Soomro, R. A. & Xu, B. Flexible carbon dots-intercalated MXene film electrode with outstanding volumetric performance for supercapacitors. *Adv. Funct. Mater.* **33**, 2209918 (2022).

16. Acerce, M., Voiry, D. & Chhowalla, M. Metallic 1T phase MoS_2 nanosheets as supercapacitor electrode materials. *Nat. Nanotechnol.* **10**, 313−318 (2015).

17. Jeon, H., *et al.* Scalable water-based production of highly conductive 2D nanosheets with ultrahigh volumetric capacitance and rate capability. *Adv. Energy Mater.* **8**, 1802691 (2018).

18. Ke, Q., *et al.* Strong charge transfer at 2H-1T phase boundary of MoS_2 for superb high-performance energy storage. *Small* **15**, 1900131 (2019).

19. Bo, Z., *et al.* Ultrathick MoS_2 films with exceptionally high volumetric capacitance. *Adv. Energy Mater.* **12**, 2103394 (2022).

20. Ma, H., *et al.* Disassembly-reassembly approach to RuO_2/graphene composites for ultrahigh volumetric capacitance supercapacitor. *Small* **13**, 1701026 (2017).

21. Li, H., Li, X., Liang, J. & Chen, Y. Hydrous RuO_2-decorated MXene coordinating with silver nanowire inks enabling fully printed micro-supercapacitors with extraordinary volumetric performance. *Adv. Energy Mater.* **9**, 1803987 (2019).

22. Zhang, Y., *et al.* Inverse opaline metallic membrane addresses the tradeoff between volumetric capacitance and areal capacitance of supercapacitor. *Adv. Energy Mater.* **12**, 2102802 (2021).

23. Li, Y.-Q., Shi, X.-M., Lang, X.-Y., Wen, Z., Li, J.-C. & Jiang, Q. Remarkable improvements in volumetric energy and power of 3D MnO_2 microsupercapacitors by tuning crystallographic structures. *Adv. Funct. Mater.* **26**, 1830−1839 (2016).

24. Lu, M., *et al.* Intercalation and delamination behavior of $Ti_3C_2T_x$ and $MnO_2/Ti_3C_2T_x$/rGO flexible fibers with high volumetric capacitance. *J. Mater. Chem. A* **7**, 12582−12592 (2019).

25. Yin, Q., Jia, H., Liu, G. & Ji, Q. Tailoring the mechanical performance of carbon nanotubes buckypaper by aramid nanofibers towards robust and compact supercapacitor electrode. *Adv. Funct. Mater.* **32**, 2111177 (2022).

26. Jo, C., *et al.* Block-copolymer-assisted one-pot synthesis of ordered mesoporous WO_{3-x}/carbon

nanocomposites as high-rate-performance electrodes for pseudocapacitors. *Adv. Funct. Mater.* **23**, 3747−3754 (2013).

27. Zhou, Y., Ko, S., Lee, C. W., Pyo, S. G., Kim, S.-K. & Yoon, S. Enhanced charge storage by optimization of pore structure in nanocomposite between ordered mesoporous carbon and nanosized WO_{3-x}. *J. Power Sources* **244**, 777−782 (2013).
28. Li, K. R., *et al.* Aluminum-ion-intercalation supercapacitors with ultrahigh areal capacitance and highly enhanced cycling stability: Power supply for flexible electrochromic devices. *Small* **13**, 1700380 (2017).
29. Zhi, J., Zhou, M., Zhang, Z., Reiser, O. & Huang, F. Interstitial boron-doped mesoporous semiconductor oxides for ultratransparent energy storage. *Nat. Commun.* **12**, 445 (2021).
30. Xiao, X., *et al.* Salt-templated synthesis of 2D metallic MoN and other nitrides. *ACS Nano* **11**, 2180−2186 (2017).
31. Robert, K., *et al.* Novel insights into the charge storage mechanism in pseudocapacitive vanadium nitride thick films for high-performance on-chip micro-supercapacitors. *Energy Environ. Sci.* **13**, 949−957 (2020).
32. Li, Q., *et al.* Spatially confined synthesis of vanadium nitride nanodots intercalated carbon nanosheets with ultrahigh volumetric capacitance and long life for flexible supercapacitors. *Nano Energy* **51**, 128−136 (2018).
33. Feng, D., *et al.* Robust and conductive two-dimensional metal-organic frameworks with exceptionally high volumetric and areal capacitance. *Nat. Energy* **3**, 30−36 (2018).
34. Wang, C., *et al.* A graphene-covalent organic framework hybrid for high-performance supercapacitors. *Energy Stor. Mater.* **32**, 448−457 (2020).

35. Xiao, K., Jiang, D., Amal, R. & Wang, D. W. A 2D conductive organic-inorganic hybrid with extraordinary volumetric capacitance at minimal swelling. *Adv. Mater.* **30**, 1800400 (2018).

36. Li, Z., *et al.* Tuning the interlayer spacing of graphene laminate films for efficient pore utilization towards compact capacitive energy storage. *Nat. Energy* **5**, 160−168 (2020).

37. Zhong, J., Sun, W., Wei, Q., Qian, X., Cheng, H. M. & Ren, W. Efficient and scalable synthesis of highly aligned and compact two-dimensional nanosheet films with record performances. *Nat. Commun.* **9**, 3484 (2018).

38. Lian, G., *et al.* Ultrafast molecular stitching of graphene films at the ethanol/water interface for high volumetric capacitance. *Nano Lett.* **17**, 1365−1370 (2017).

39. Xu, Y., *et al.* A metal-free supercapacitor electrode material with a record high volumetric capacitance over 800 F cm^{-3}. *Adv. Mater.* **27**, 8082−8087 (2015).

40. Wang, Y., Yang, X., Pandolfo, A. G., Ding, J. & Li, D. High-rate and high-volumetric capacitance of compact graphene-polyaniline hydrogel electrodes. *Adv. Energy Mater.* **6**, 1600185 (2016).

41. Fan, Z., Zhu, J., Sun, X., Cheng, Z., Liu, Y. & Wang, Y. High density of free-standing holey graphene/ppy films for superior volumetric capacitance of supercapacitors. *ACS Appl. Mater. Interfaces* **9**, 21763−21772 (2017).

42. Dong, X., *et al.* High volumetric capacitance, ultralong life supercapacitors enabled by waxberry-derived hierarchical porous carbon materials. *Adv. Energy Mater.* **8**, 1702695 (2018).

43. Zhang, G., Guan, T., Qiao, J., Wang, J. & Li, K. Free-radical-initiated strategy aiming for pitch-based dual-doped carbon nanosheets engaged into high-energy asymmetric supercapacitors. *Energy Stor. Mater.* **26**, 119−128 (2020).

44. Yan, L., *et al.* N, P, S-codoped hierarchically porous carbon spheres with well-balanced

gravimetric/volumetric capacitance for supercapacitors. *ACS Sustainable Chem. Eng.* **6**, 5265−5272 (2018).

45. Huang, J., Zhang, W., Huang, H., Liu, Y., Yang, Q. & Li, L. Facile synthesis of N, S-codoped hierarchically porous carbon with high volumetric pseudocapacitance. *ACS Sustainable Chem. Eng.* **7**, 16710−16719 (2019).

46. Zhang, S.*, et al.* Ultramicroporous carbons puzzled by graphene quantum dots: Integrated high gravimetric, volumetric, and areal capacitances for supercapacitors. *Adv. Funct. Mater.* **28**, 1805898 (2018).

47. Wang, G.*, et al.* A high-capacity negative electrode for asymmetric supercapacitors based on a PMO12 coordination polymer with novel water-assisted proton channels. *Small* **16**, 2001626 (2020).

48. Kandambeth, S.*, et al.* Covalent organic frameworks as negative electrodes for high-performance asymmetric supercapacitors. *Adv. Energy Mater.* **10**, 2001673 (2020).

49. Wang, G.*, et al.* POMCPs with novel two water-assisted proton channels accommodated by MXenes for asymmetric supercapacitors. *Small* **18**, 2202087 (2022).

50. Zhang, Y.*, et al.* In-situ synergistic $W_{18}O_{49}/Ti_3C_2T_x$ heterostructure as negative electrode for high energy density supercapacitors. *Carbon* **208**, 92−101 (2023).

51. Patil, A. M.*, et al.* Bilateral growth of monoclinic WO_3 and 2D $Ti_3C_2T_x$ on 3D free-standing hollow graphene foam for all-solid-state supercapacitor. *Chem. Eng. J.* **421**, 127883 (2021).

52. Cui, L., Yu, K., Lv, J., Guo, C. & Zhou, B. A 3D POMOF based on a {AsW12} cluster and a Ag-MOF with interpenetrating channels for large-capacity aqueous asymmetric supercapacitors and highly selective biosensors for the detection of hydrogen peroxide. *J. Mater. Chem. A* **8**, 22918−22928 (2020).

53. Shi, M., Peng, C. & Zhang, X. A novel aqueous asymmetric supercapacitor based on pyrene-4,5,9,10-tetraone functionalized graphene as the cathode and annealed $Ti_3C_2T_x$ MXene as the anode. *Small* **19**, 2301449 (2023).

54. Luo, Y., Yang, C., Tian, Y., Tang, Y., Yin, X. & Que, W. A long cycle life asymmetric supercapacitor based on advanced nickel-sulfide/titanium carbide (MXene) nanohybrid and MXene electrodes. *J. Power Sources* **450**, 227694 (2020).

55. Tomar, A. K., Singh, G. & Sharma, R. K. Charge storage characteristics of mesoporous strontium titanate perovskite aqueous as well as flexible solid-state supercapacitor cell. *J. Power Sources* **426**, 223−232 (2019).

56. Tomar, A. K., Kshetri, T., Kim, N. H. & Lee, J. H. Cation and anion (de)intercalation into MXene/perovskite oxides for high-rate intercalation pseudocapacitance. *Energy Stor. Mater.* **50**, 86−95 (2022).

57. Tang, X., Zhang, B., Lui, Y. H. & Hu, S. Ni-Mn bimetallic oxide nanosheets as high-performance electrode materials for asymmetric supercapacitors. *J. Energy Storage* **25**, 100897 (2019).
