# Supplementary material for: Discovery of fast and stable proton storage in bulk hexagonal molybdenum oxide
Source: Nat Commun. 2023 Dec 15;14:8360. doi: 10.1038/s41467-023-43603-6 (PMC10724264; doi:10.1038/s41467-023-43603-6)
Supplement: Supplementary file 3 — Description of Additional Supplementary Files [file 41467_2023_43603_MOESM3_ESM.pdf]

Description of Additional Supplementary Files

Supplementary Movie 1

The migration and coordination processes of different protons in h-$MoO_3 \cdot 0.7H_2O$.
